# Change in Body Weight and Serum Albumin Levels in Febrile Neutropenic Lung Cancer Patients

**DOI:** 10.31372/20200503.1106

**Published:** 2020

**Authors:** Naomi Kayauchi, Yumi Nakagawa, Takako Oteki, Katsunori Kagohashi, Hiroaki Satoh

**Affiliations:** University of Tsukuba, Mito Medical Center, Japan

**Keywords:** nutrition, total protein, albumin, body weight, febrile neutropenia, lung cancer, nursing

## Abstract

Although advances have been made in the treatment and prevention of febrile neutropenia (FN) in cancer patients treated with chemotherapy, it is still a complication that requires clinical attention. Impaired nutritional status in patients who develop FN can affect the continuation of cancer treatment, but it has not been investigated. We conducted a retrospective longitudinal study in order to clarify (1) if body weight and serum albumin levels change in lung cancer patients who do and do not develop FN, and (2) if these indicators are more likely to worsen in patients with FN than in patients without FN. Patients undergoing cytotoxic chemotherapy between January 2011 and June 2020 were consecutively included in the study. Changes in body weight and serum albumin levels were investigated in a case–control study of patients with FN, and control patients without FN who were matched by age, gender, histopathology, and stage of lung cancer, at a ratio of 1:2. During the study period, 226 patients received cytotoxic chemotherapy. Among those, 33 (14.6%) patients developed FN during the first course of cytotoxic chemotherapy. We found a more pronounced decrease in both body weight and serum albumin level at four weeks after the initiation of chemotherapy in FN patients. In order to safely administer effective chemotherapy, medical staff need to pay close attention to the nutritional status of patients receiving chemotherapy.

## Introduction

Febrile neutropenia (FN) is a medical emergency defined as fever in patients with abnormally low number of circulating neutrophils, commonly associated with cytotoxic chemotherapy (Crawford, Caserta, & Roila, 2010; Freifeld et al., 2010; NCCN, 2012; Smith et al., 2006). There have been several reported indicators of FN, including body temperature, duration of fever, and decreased neutrophil counts (Crawford et al., 2010; Freifeld et al., 2010; NCCN, 2012; Smith et al., 2006). Whichever indicators are used, it is a complication that should be noted, because it may lead to a deterioration of the general condition, and delay or difficulty of in the continuation of chemotherapy. In chemotherapy for lung cancer, FN develops in 3.7–28% of patients (Smith et al., 2006), and therefore, FN is not a rare complication. With the advent of granulocyte-colony stimulating factor (G-CSF) preparations, progress has been made in the treatment and prevention of FN, but it is still a complication requiring clinical attention (Petru et al., 2015). Pretreatment weight loss (underweight) and hypoalbuminemia have been reported to be two of the important risk factors of the development of FN in several hematological and nonhematological malignant diseases (Aoyagi, Morii, Tajima, Yoshiyama, & Ichimura, 2015; Kobayashi et al., 2019; Lapointe et al., 2005; Pettengell et al., 2009; Schwenkglenks, Bendall, Pfeil, Szabo, & Pettengell, 2013). Studies such as the association of the nutritional status and the quality of life (Citak, Tulek, & Uzel, 2019), the prevention of weight loss by G-CSF administration (Pettengell et al., 2009), and the role of low bacterial diet in FN (Heng, Barbon Gauro, Yaxley, & Thomas, 2020; van Dalen et al., 2016) have been reported.

Many countries in the Asian and American Pacific Islands have high lung cancer mortality rates due to still high smoking rates and the aging of the population (Cheng et al., 2014; Didkowska, Wojciechowska, Mańczuk, & Łobaszewski, 2016; Torre et al., 2016). In addition, many clinical trials of lung cancer chemotherapy are being conducted as global researches, and patient registration from many Asian countries is indispensable. Under these circumstances, there is an increasing demand for nurses involved in decision making in team medical care, even if they are not certified nurse specialists. Accumulation of knowledge on the management of complications associated with chemotherapy and feedback on its practice are important. From this point of view, it is evaluated that this study may contribute to the progress of nursing science.

In both lung cancer patients and those with malignant diseases other than lung cancer, to our best knowledge, however, no studies have been conducted comparing changes in body weight and serum albumin levels between patients with and without FN. The purpose of this study was to clarify (1) how body weight and serum albumin levels change in lung cancer patients with and without FN, and (2) whether these indicators are more likely to worsen in lung cancer patients with FN than those without FN. To clarify these questions, we conducted a case–control study of lung cancer patients who did or did not develop FN during cytotoxic chemotherapy.

## Methods

### Study Settings and Samples

This study had a longitudinal retrospective, nonexperimental case–control design. We consecutively investigated medical records in all the lung cancer patients who were treated with cytotoxic chemotherapy in a tertiary hospital in Japan. Lung cancer was pathologically categorized according to the World Health Organization classification. A tumor-node-metastasis staging procedure (8th edition, TNM Classification) using head computed tomography (CT) or magnetic resonance imaging, bone scans and ultrasonography and/or CT of the abdomen was performed for all patients prior to starting treatment.

We listed all lung cancer patients who received chemotherapy from the institutional database between January 2011 and June 2020, and investigated all patients who were treated with first-course of the first-line cytotoxic chemotherapy during the study period. Criteria were prepared to exclude patients who lacked information on patient characteristics, pre- and post-treatment body weights and serum albumin levels, and who died within 4 weeks of the start of the first course of first chemotherapy. However, no patient met this exclusion criterion. It was unquestioned whether the second and subsequent courses of chemotherapy could be continued.

### Instruments

Changes in body weight and serum level of albumin were investigated in a case–control study of lung cancer patients who developed FN during the first course of cytotoxic chemotherapy and control patients who did not develop FN. Control patients were matched to FN patients by age, gender, histopathology, and stage of lung cancer at a ratio of 2:1. With reference to some guidelines (Crawford et al., 2010; Freifeld et al., 2010; NCCN, 2012; Smith et al., 2006), FN was defined as fever (38 °C or higher) in patients whose neutrophil count was less than 1,000/μL due to chemotherapy.

### Data Collection Procedures

Age, gender, histology of lung cancer, clinical stage, and chemotherapy regimen administered were retrospectively investigated in the medical records of each patient with or without FN. Pretreatment body weight and serum level of albumin and those at the time of 4 weeks after the initiation of the first course of the first-line chemotherapy were retrospectively investigated in the medical records of each patient with or without FN at the time of initiation of the first course of the first-line cytotoxic chemotherapy and four weeks thereafter.

### Data Analysis Methods

Retrospective chart review during the study period identified 33 lung cancer patients who developed FN and 193 patients without FN. Propensity score matching was used to adjust for baseline differences between patients with and without FN (D’Agostino, 1998). The variables included in a logistic regression analysis as predictors were age, sex, histological type, disease stage, and chemotherapy regimen. Due to the small number of FN patients, patients without FN were set to be assigned in a 1:2 ratio using propensity scores generated from the analysis. This sample size was considered adequate based on the power analysis (Cohen, 1988). According to the power analysis conducted with GPower version 3.1 (Faul, Erdfelder, Buchner, & Lang, 2009), the minimum sample size required was 78 (*α* = 0.05, size = 0.13, power = 0.80). Effect size (0.13) was derived from a study by Lindvig et al. (2014). From the 193 patients without FN, 65 control patients, who were matched by age, sex, histopathology, stage of lung cancer, and chemotherapy regimen were selected. Demographic and clinical characteristics were reported for patients with FN and control patients without FN separately. Categorical variables were reported as counts and percentages. Continuous variables were summarized as medians and standard deviations. Chi-square tests and Mann–Whitney *U* test were used to calculate potential differences in these distributions between the patients with and without FN. The Wilcoxon signed-rank sum test was also used for comparison of the all-cause cost and the inpatient cost variables. All analyses were performed using SPSS version 23 (IBM Corporation, Armonk, New York). A critical value of 0.05 was specified a priori as the threshold for statistical significance.

### Ethics

This retrospective noninterventional study conformed to the Declaration of Helsinki and the Ethical Guidelines for Clinical Studies issued by the Ministry of Health, Labor and Welfare in Japan. Written comprehensive consent for a noninterventional retrospective study was obtained from each patient. Analysis of the medical records of lung cancer patients was approved by the ethics committee in our institute.

## Results

### Patient Characteristics

**Figure 1.**
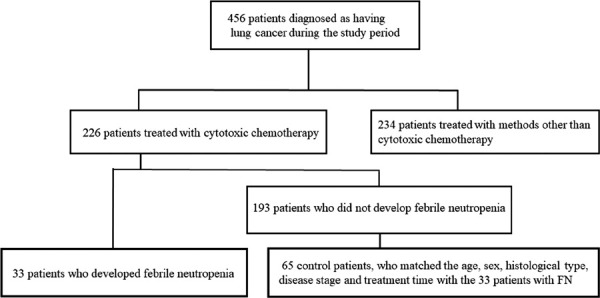
Study flow chart.

During the study period, 456 patients were diagnosed with lung cancer. Among them, 226 patients received cytotoxic chemotherapy, and of these, 33 (14.6%) developed FN (Figure 1). Table 1 shows the characteristics of the 33 patients with FN and the 193 patients without FN. There were no significant factors associated with the development of FN. From the control patients without FN, we matched 65 patients with the 33 patients with FN by age, sex, histological type, disease stage, and treatment time at a ratio of 2:1. The characteristics of these two groups of patients are shown in Table 2.

**Table 1:** Characteristics in Lung Cancer Patients With and Without Febrile Neutropenia

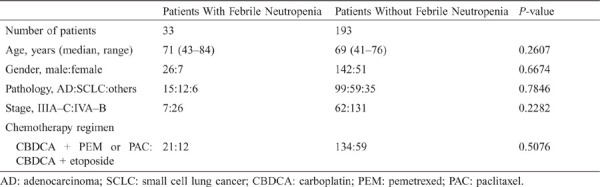

**Table 2:** Characteristics in Lung Cancer Patients with Febrile Neutropenia, and Control Patients Without Febrile Neutropenia who were Case-Matched by Age, Gender, Histopathology, and Stage of Lung Cancer at a Ratio of 1:2

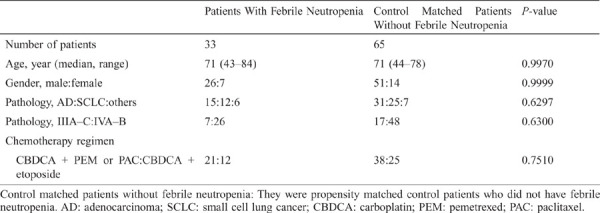

### Body Weight and Serum Albumin Level Changes

**Figure 2.**
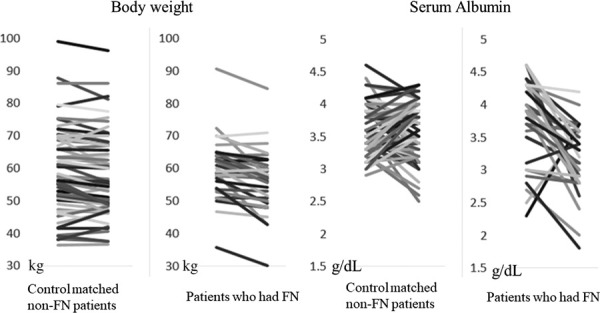
Comparison of body weight and serum albumin level at the time of initiation of chemotherapy (left) and at four weeks after the initiation of treatment (right) in patients who had FN and control matched non-FN patients.

In patients with FN, body weight and serum albumin levels decreased four weeks after the initiation of chemotherapy (*P* = 0.0001, respectively) (Figure 2). In patients without FN, weight loss was significant (*P* = 0.0403), but serum albumin reduction was not (*P* = 0.1051).

### Body Weight and Serum Albumin Levels Before and Four Weeks After the Initiation of Chemotherapy

Between patients with FN and control patients without FN, there was no difference in body weight and serum albumin level at the time of initiation of chemotherapy (*P* = 0.3750 and *P* = 0.1051, respectively) (Table 2). After four weeks, body weight did not differ significantly (*P* = 0.9820). However, in FN patients, serum albumin level four weeks after the initiation of chemotherapy was significantly lower than that at initiation (*P* = 0.0403).

### Changes in Body Weight and Serum Levels of Albumin Before and Four Weeks After the Initiation of Chemotherapy

Table 3 shows the changes in body weight and serum albumin levels before and four weeks after the initiation of chemotherapy. The decreases in both body weight and serum albumin levels four weeks after the initiation of chemotherapy were more marked in FN patients than in control patients without FN (*P* = 0.0027 and *P* = 0.0001, respectively).

**Table 3:** Clinical Characteristics of Patients with Febrile Neutropenia and Control Patients Without Febrile Neutropenia, who were Case-Matched by Age, Gender, Histopathology, and Stage of Lung Cancer at a Ratio of 1:2

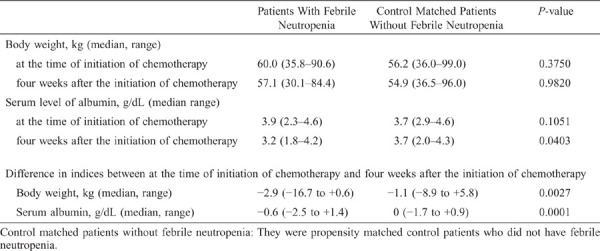

## Discussion

According to a review, FN occurred at a frequency of 5.8–14.6% in colorectal cancer patients, 3.7–28% in lung cancer patients, 2–34% in breast cancer patients, and 18–48% in patients with malignant lymphoma (Smith et al., 2006). The frequency of FN might change depending on the antineoplastic drug regimen administered, and the frequency of FN might be decreasing due to the clinical uptake of G-CSF therapy. However, there is no doubt that FN is still a complication that cannot be overlooked clinically. Good nutritional status before treatment is important when undergoing chemotherapy (Chlebowski, 1985). Serum total protein and albumin levels are used as indicators of nutritional status (Sasatani, Okauchi, Ohara, Kagohashi, & Satoh, 2020; Shen et al., 2020). Several studies have used body weight and BMI as indicators of general condition in patients receiving chemotherapy (Castillo-Martínez et al., 2018). Furthermore, pretreatment weight loss is a poor prognostic factor in lung cancer patients (Lau et al., 2018). In recent years, the concepts of cachexia, sarcopenia, and frailty have become widespread, and studies based on these concepts have been conducted in the area of cancer treatment (Jain, Coss, Whooley, & Phelps, 2020; Kidd, Skrzypski, Jamal-Hanjani, & Blyth, 2019; Kurishima, Watanabe, Ishikawa, Satoh, & Hizawa, 2017; Nakamura et al., 2018; Phillips, Hug, Allan, & Ezhil, 2019). We have also been interested in cachexia and sarcopenia in cancer patients, and have reported some findings (Kurishima et al., 2017; Nakamura et al., 2018). We have reported, not only on the condition of patients before treatment, but also on the changes in nutritional status during the course of treatment in patients with chronic respiratory disease (Sasatani et al., 2020). In lung cancer patients who developed FN, several factors have been associated with the development of FN (Cupp et al., 2018; Pawloski et al., 2017). To our knowledge, however, changes in weight and nutritional status of lung cancer patients who develop FN have not been reported.

At present, many clinical trials of lung cancer chemotherapy are being conducted as global studies, and patient registration from Asian countries is indispensable. Due to high smoking prevalence and an aging population, in addition, many countries in the Asian and Pacific Islands have high lung cancer mortality rates (Cheng et al., 2014; Didkowska et al., 2016; Torre et al., 2016). Under these circumstances, there is an increasing demand for nurses involved in decision making in team medical care, even if they are not certified nurse specialists. Accumulation of knowledge about managing the complications associated with chemotherapy and feedback on its practice are important. From this point of view, this research is evaluated to contribute to the progress of nursing science.

We could not find direct biological evidence as to why hypoalbuminemia and weight loss were the factors that caused the development of FN. In the condition of undernutrition, there are disruption of defense against invasion of pathogens (Knaus, Hertzberger, Pircalabioru, Yousefi, & Branco Dos Santos, 2017), decrease in production and replacement of substances and immune cells that are responsible for cellular and humoral immunity (Amati et al., 2003). As a result, undernourished patients might be at increased risk of developing FN. From the results of this study, attention should also be paid to post-treatment malnutrition in FN patients.

There are two most common FN risk index scoring system recommended by the guidelines. One is the Multinational Cancer Supportive Care Association (MCSCA) scores provided by The ASCO Guidelines. Another one is the clinical index of stable febrile neutropenia score (CISNE) by Supportive Care Working Group of the Spanish Society of Medical Oncology (Carmona-Bayonas et al., 2017). In both MCSCA and CISNE, however, malnutrition was not included in these factors (Carmona-Bayonas et al., 2017; Klastersky et al., 2000).

In the present study, 14.9% of patients had FN in the first course of cytotoxic chemotherapy. This result confirms that FN is a possible complication in chemotherapy for lung cancer that must be paid attention to. It is open to debate whether G-CSF should be administered from the first course of chemotherapy, but it seems that we were able to provide reference information for this discussion as well. It was considered that the nutritional status before treatment might be related to the onset of FN. However, in this study, there was no significant relationship between body weight and serum albumin level at the initiation of chemotherapy and the development of FN. Chemotherapy for lung cancer is usually administered every three to four weeks. Taking this into consideration, this study compared body weight and serum albumin levels at the initiation of chemotherapy and four weeks thereafter. In patients with FN, there was a significant decrease in body weight and serum albumin levels at four weeks after the initiation of chemotherapy. Moreover, the decreases were more pronounced in patients with FN compared with those without FN. Thus, it appeared that nutritional status deteriorated due to the onset of FN. The results of this study can provide direction for FN prevention and appropriate treatment for FN.

Despite some interesting findings, this study had some limitations. First, this was a retrospective study within a single institute, and included a comparatively small number of patients. Second, our patients were treated with different chemotherapy regimens. Third, detailed information on patient comorbidities was lacking in the records. Fourth, there were no analyses concerning the continuation of chemotherapy in patients with FN. We could not perform analyses that included information about response to chemotherapy, and continuation of chemotherapy in patients with FN. However, this study represented the current state of our medical care, and might provide useful information on the change in nutritional status of lung cancer patients who develop FN, and to plan future research in this area.

## Conclusion

Lung cancer patients who develop FN are more likely to have a reduced nutritional status after chemotherapy. Based on the study findings, we recommended that medical staffs need to pay close attention to the changes in body weight and serum level of albumin after the initiation of chemotherapy in patients with FN. Studies will be required to investigate the effects of these changes on chemotherapy continuation, chemotherapy response, and prolongation of survival.

## Declaration of Conflicting Interests

The authors declared no potential conflicts of interest concerning the research, authorship, or publication of this article.

## Ethical Considerations

This study conformed to the Ethical Guidelines for Clinical Studies issued by the Ministry of Health, Labor and Welfare of Japan. Written comprehensive consent for a noninterventional retrospective study was obtained from each patient. Analysis of the medical records of lung cancer patients was approved by the Ethics Committee in Mito Medical Center, University of Tsukuba Hospital (NO 16–19).

## Authorship Statement

All authors met the authorship criteria, and all authors were in agreement with the content of the manuscript.
